# Multi-scale Characterisation of the 3D Microstructure of a Thermally-Shocked Bulk Metallic Glass Matrix Composite

**DOI:** 10.1038/srep18545

**Published:** 2016-01-04

**Authors:** Wei Zhang, Andrew J. Bodey, Tan Sui, Winfried Kockelmann, Christoph Rau, Alexander M. Korsunsky, Jiawei Mi

**Affiliations:** 1 School of Engineering, University of Hull, Hull, HU6 7RX, East Yorkshire, UK; 2 Diamond Light Source, Oxfordshire, OX11 0DE, UK; 3 Multi-Beam Laboratory for Engineering Microscopy (MBLEM), Department of Engineering Science, University of Oxford, Parks Road, Oxford, OX1 3PJ, UK; 4 ISIS Neutron and Muon Source, Rutherford Appleton Laboratory, Oxfordshire, OX11 0QX, UK

## Abstract

Bulk metallic glass matrix composites (BMGMCs) are a new class of metal alloys which have significantly increased ductility and impact toughness, resulting from the ductile crystalline phases distributed uniformly within the amorphous matrix. However, the 3D structures and their morphologies of such composite at nano and micrometre scale have never been reported before. We have used high density electric currents to thermally shock a Zr-Ti based BMGMC to different temperatures, and used X-ray microtomography, FIB-SEM nanotomography and neutron diffraction to reveal the morphologies, compositions, volume fractions and thermal stabilities of the nano and microstructures. Understanding of these is essential for optimizing the design of BMGMCs and developing viable manufacturing methods.

Since the 1980s^1^, bulk metallic glasses (BMGs) have attracted much attention from the physics, materials and engineering communities because of their exceptionally high strength, elastic limits, as well as excellent corrosion and wear-resistance^2^. However, most BMGs have very limited ductility, and often exhibit brittle fracture under tensile loads^3^,^4^. Extensive studies have been carried out in the past twenty years to increase the ductility of BMGs^5^. One of the most promising approaches is to design BMG-based composites in which ductile crystalline phases nucleate *in situ* from the super-cooled liquid, and then grow to form ductile dendrites distributed uniformly in the amorphous matrix. Such bulk metallic glass matrix composites (BMGMCs) have significantly increased impact toughness and large strain to failure in tension and compression^6^. Among the few BMGMCs developed to date, the Zr-Ti based system (DH1-3) is one of the successful examples^6^. Figure 1(b) shows the typical microstructure of a Zr_39.6_Ti_33.9_Nb_7.6_Cu_6.4_Be_12.5_ (atomic mass %) alloy (DH3) with crystalline dendritic structures uniformly distributed throughout the amorphous matrix.

A recent *in situ* deformation study using synchrotron X-ray diffraction and electron microscopy imaging revealed that the dendrites - and particularly the strongly-bonded interfaces between the dendrites and the amorphous matrix - play a crucial role in enhancing the ductility of the composite^7^. The morphology, size, volume fraction and distribution of the dendrites are the key factors that control the mechanical properties of the composite^6^. However, almost all previous characterization studies of these composites have been performed on two dimensional (2D) sections using electron microscopy. Focused ion beam milling plus scanning electron microscopy (FIB-SEM) is a standard technique to generate 3D reconstructions through a series of sectioning and imaging. For example, Xu *et al.* used this method to study the 3D morphology of Mg flakes in a Mg-based BMG composite^8^. However, FIB-SEM is practically limited to the sectioning of sub-micrometre structural features, and is therefore not suitable for studying the dendrites in BMGMCs, which are generally tens or hundreds of microns in length.

Applying pulsed electric currents with high current density into bulk metallic glasses can accelerate the diffusion and rearrangement of solute atoms over a short period of time^9^,^10^, and therefore promote nanocrystallisation. Johnson *et al.*^11^ and Liu *et al.*^12^ found that very rapid heating (up to 10^6^ K/s) induced by electric current pulse can be used to heat BMGs into the supercooled liquid state without causing crystallisation; in this state, near net-shape thermoplastic processing can be applied. Johnson *et al.*^11^ also argued that most BMG systems have homogeneous and virtually temperature-independent electrical resistivities, so that passing an electric current pulse through the bulk samples can generate uniform volumetric Joule heating for the entire sample volume. In addition, Lee *et al.*^13^ studied the thermal stability of the LM2A2 at low temperature (around 300 °C in isothermal conditions), and found that the body centred cubic (BCC) dendritic β-Zr phase is unstable below 300 °C because Zr tends to form the hexagonal close packed (HCP) crystal structure in low temperature. They also found that the BCC β-Zr phase is stable at high temperatures (~600 °C), while the amorphous matrix crystallises at this temperature during isothermal heat treatment. No studies have been published concerning the influence of high density electric currents on the nano/microstructure evolution of BMGMCs, in which different phases have different electrical resistivities. Hence, two main scientific questions that remain to be answered are (1) the thermal stability of the different phases in BMGMCs under rapid Joule heating induced by high density electric currents, (2) the crystallisation behavior of the amorphous matrix within the BMGMCs when compared to that of monolithic BMG.

In this paper, we report the use of a Gleeble 3500 thermo-mechanical simulator to apply high density electric currents to rapidly heat a DH3 BMGMC to different preset temperatures. We then used neutron diffraction to characterise the phase changes in the bulk samples, and synchrotron X-ray microtomography to characterise the 3D morphologies of the crystalline dendrites. Between the crystalline dendrites, the nanostructures were further analysed using FIB-SEM serial sectioning and 3D reconstruction. The thermal stability of the DH3 BMGMCs under thermal shock induced by high density electric current, and the corresponding 3D structure of the crystalline dendrites, the amorphous matrix and the newly formed phases due to different thermal shock conditions are also presented in this paper.

## Results

**Figure 1:**
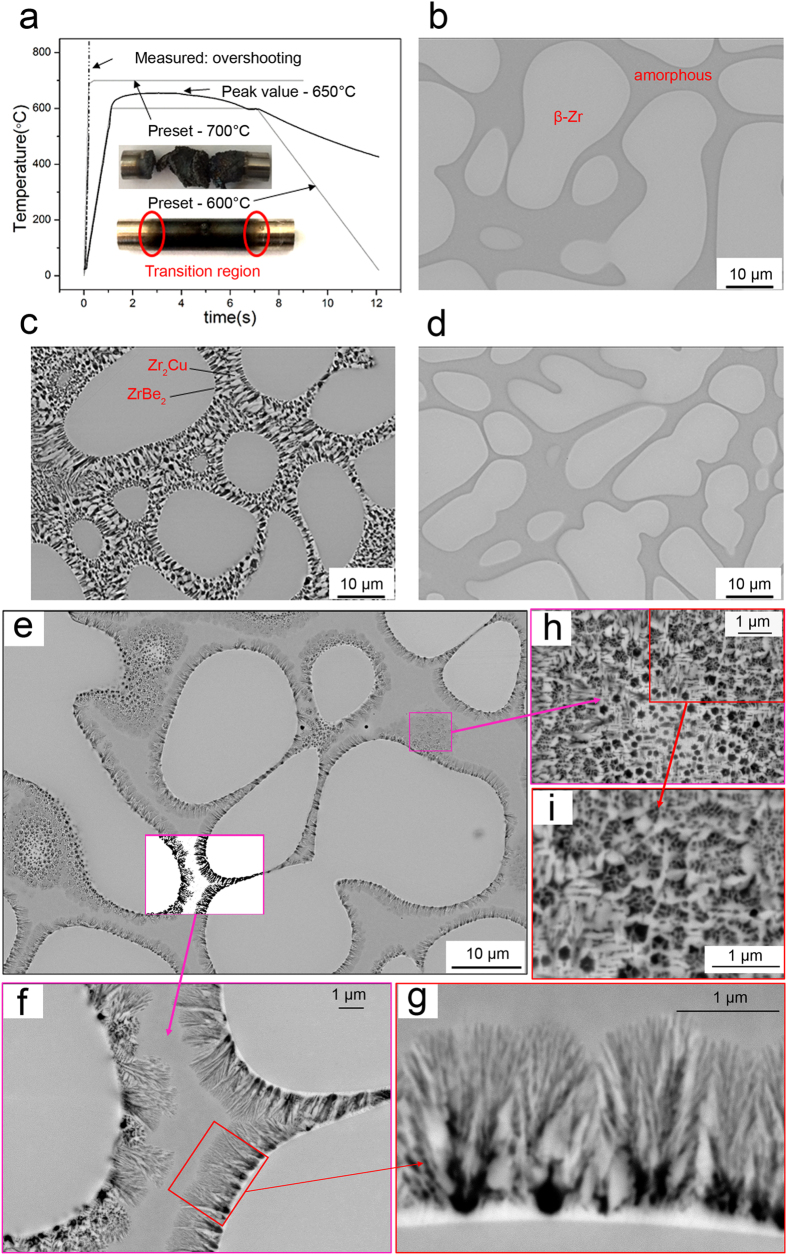
The thermal shock temperature profiles and the SEM micrographs of the as-cast and thermally shocked DH3. (**a**) The preset and measured temperatures for the thermally-shocked samples. Typical scanning electron microscopy (SEM) micrographs for (**b**) the as-cast sample; and the samples thermally shocked to (**c**) 650 °C (600 °C preset), and (**d**) to the point of melting (700 °C preset). (**e**) Eutectic growth of the needle phases Zr_2_Cu (dark) and ZrBe_2_ (light) found in the transition zone between the thermally shocked region and the as-cast region for the sample shocked to 650 °C (marked by a red oval in (**a**)). (**f**,**g**) are the enlarged magnification of the framed areas in (**e**,**f**) respectively. (**h**,**i**) represent the higher magnification of the framed areas in (**e**,**h**) respectively, illustrating the intermetallic phases which nucleated directly from the amorphous matrix.

Figure 1a shows the preset and measured temperature profiles and photographs of the two round bar samples after the Gleeble thermal shock experiments. For the sample preset to 600 °C, the temperature at the central region reached 650 °C. The sample preset to 700 °C was completely melted in the middle due to the temperature overshoot; the thermocouple positioned in the middle was therefore not able to record the actual temperature profile.

Figure 1b shows a typical backscatter scanning electron micrograph for the as-cast sample. Dendrites (light regions with composition of Zr_43.2_Ti_41.1_Nb_14.3_Cu_1.4,_ often called β-Zr) are uniformly distributed in the amorphous matrix (Zr_32.1_Ti_18.8_ Nb_3.3_Cu_9.8_Be_36.0_^6^). For the sample thermally shocked to 650 °C, two more phases (light and dark) are visible in Fig. 1c. Figure 1e–g show that the new phases that nucleated at the dendrite-amorphous matrix interface grew into the amorphous matrix until it was almost completely consumed (Fig. 1c). However, no noticeable change was found for the dendritic phase. The fact that the amorphous matrix transformed into two other crystalline phases in just 6 s of thermal shock processing indicates that the primary dendritic crystalline phase is more thermodynamically stable than the amorphous matrix^4^. In fact, the dendrites have electric resistivities (~50 *μΩ*·*cm*), much lower than that of the amorphous matrix^11^ (~250 *μΩ*·*cm*). Hence, there was less Joule heat generated in the crystalline phases. The difference in electric resistivity between the two phases also results in highly localized Ohmic dissipation across the dendrite-matrix interface^11^, explaining why nucleation events for the secondary crystalline phases occur predominantly at the interface.

**Figure 2:**
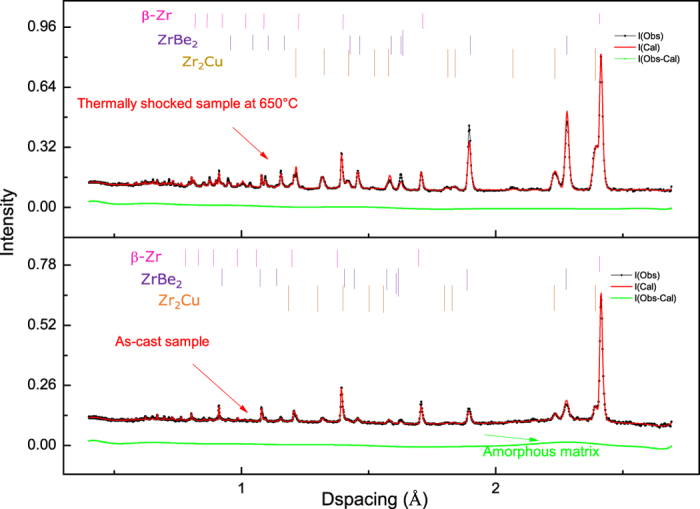
The neutron diffraction spectra and fittings for the thermally shocked and as-cast samples. Neutron diffraction spectra for (**a**) the sample thermally-shocked to 650 °C, showing profile fits for the β-Zr, ZrBe_2_, and Zr_2_Cu phases, and (**b**) the as-cast sample with fits for the β-Zr, amorphous matrix, ZrBe_2_ and Zr_2_Cu phases.

**Figure 3:**
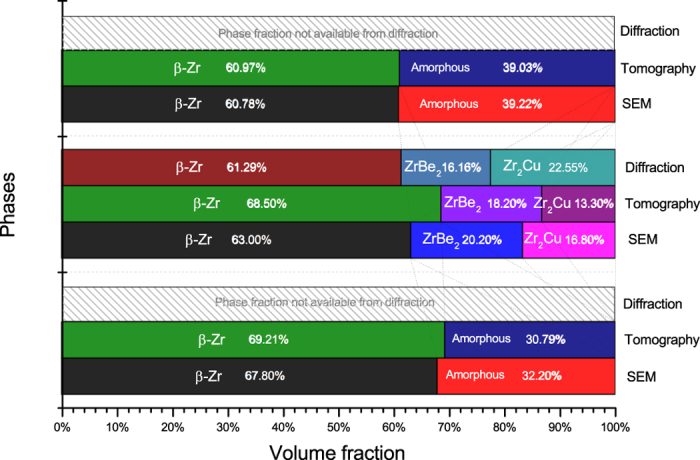
Volume fractions of β-Zr, ZrBe_2_ and Zr_2_Cu calculated from SEM 2D images, X-ray tomography and neutron diffraction.

Figure 2 shows neutron diffraction patterns for the as-cast region and 650 °C thermally-shocked region of a single rod bar. For the as-cast region, a strong β-Zr signal (magenta) was observed together with signal relating to the amorphous matrix (green traces relating to ZrBe_2_ and Zr_2_Cu). The ZrBe_2_ and Zr_2_Cu signals may arise from diffraction of the large neutron beam (15 × 15 mm) from the thermally-shocked region, because neither of these phases was observed in the SEM images of the as-cast sample. For the 650 °C thermally-shocked sample, the aforementioned three crystalline phases were also observed. Volume fractions, calculated from the fitted spectra, are summarised in Fig. 3. Much higher volume fractions of the Zr_2_Cu, and ZrBe_2_ are present in the thermally-shocked samples. The crystallisation of DH3 is different from the nanocrystallisation behaviour of Vitalloy 1 (Zr_41_Ti_14_Cu_12.5_Ni_10_Be_22.5_) BMG^14^ induced by pulsed electric current reported by Yao *et al.*^10^, which followed the sequence of amorphous → amorphous + icosahedral phase → Be_2_Zr + Zr_2_Cu + Ni_7_Zr_2_ + FCC structure phase + others → Zr_2_Cu + Ni_7_Zr_2_ + FCC structure phase + others. ZrBe_2_ and Zr_2_Cu appeared to grow together into the amorphous matrix. For the as-cast sample, it was not possible to calculate the volume fraction for the amorphous matrix from the diffraction data since amorphous material cannot be modelled crystallographically without employing auxiliary methods, such as introducing the internal or external standard to compare with the unknown amorphous phases^15^.

**Figure 4:**
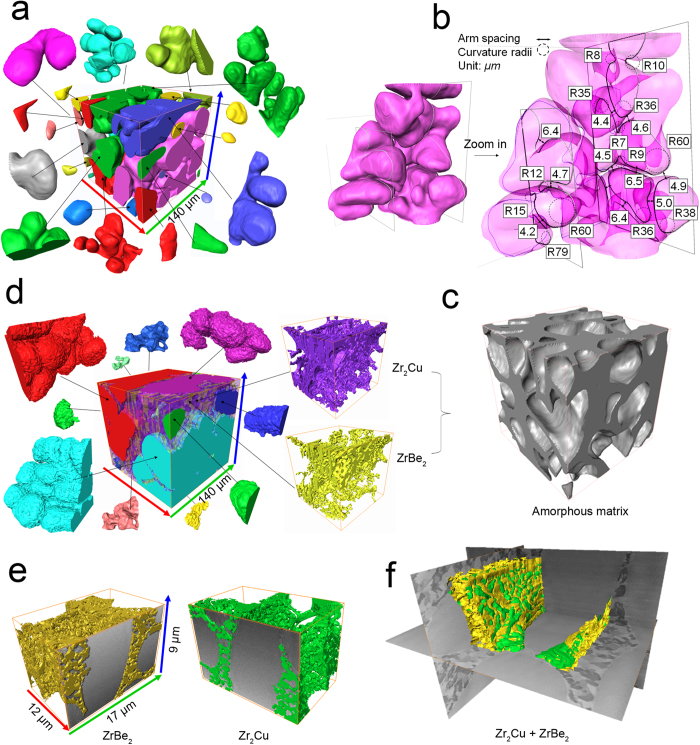
The morphologies of the phases segmented in 3D from the X-ray and FIB-SEM tomography dataset. (**a**) Primary β-Zr dendrites in the as-cast sample, with the amorphous matrix hidden for ease of visualization; (**b**) a typical dendrite and measurement of arm spacing and curvature radii; (**c**) amorphous matrix within the as-cast sample; (**d**) secondary ZrBe_2_ (yellow), Zr_2_Cu (purple) intermetallics and primary β-Zr dendrites (other colours) of the 650 °C thermally-shocked sample; (**e**,**f**) FIB-SEM sectioning nano-tomography of the 650 °C thermally-shocked sample.

Figure 4 shows the 3D microstructures obtained using synchrotron X-ray microtomography and FIB-SEM serial sectioning (more detailed 3D structure information can be seen in the Supplementary Video S1 and S2). These 3D techniques reveal information not accessible via 2D methods (e.g. SEM in Fig. 1). The 3D dendrites are actually made of many interconnected and well-developed secondary arms, as highlighted by the single dendrite coloured in magenta (Fig. 4a). As shown in Fig. 4b, the quantitative information extracted from this single dendrite indicates that most of the secondary dendrite tips have curvature radii ranging from 35 to 80 μm; their roots have curvature radii ranging from 8 to 15 μm; arm spacing ranges from 4.4 to 6.5 μm. When conventional 2D characterisation methods were used, these secondary dendrite arms were often identified as individual circular-shaped particles^16^. Each dendrite actually consists of a few to a few tens of secondary arms, with an overall size of a few hundred micrometres.

Figure 4b shows that, for the thermally shocked sample, the primary β-Zr dendrites were slightly dissolved at the dendrite-amorphous matrix interface and the dissolved layer turned into the two new phases (Zr_2_Cu and ZrBe_2_), and almost all amorphous matrix had been transformed into these two phases. However, the spatial resolution of the X-ray microtomography was insufficient to differentiate the intermetallic grains (some of which were submicron in size) clearly. We therefore used FIB-SEM nano-tomography with the effective voxel size of 25 × 25 × 50 nm to study these phases. Figure 4d–f show that the amorphous matrix was completely transformed into a highly interconnected 3D network of eutectic microstructure of Zr_2_Cu and ZrBe_2_ with a minor change of β-Zr dendrites, which is different from the amorphous matrix within the as-cast sample presented in Fig. 4c. The powerful combination of X-ray microtomography with FIB-SEM nanotomography allows us to characterise the 3D structure from nano- to micro- scale.

Volume fractions calculated for the different phases from SEM imaging, X-ray microtomography and neutron diffraction are summarized in Fig. 3. For β-Zr dendrites in the as-cast sample, results from SEM and tomography are very similar. However for the thermally shocked region, fractions from tomography for the β-Zr dendrites are ~10% higher than those obtained from neutron diffraction and SEM, and lower for the two new ZrBe_2_ and Zr_2_Cu. Phases. This is most likely due to the difficulties in segmentation of ZrBe_2_ and Zr_2_Cu phases. The grain sizes are typically just a few micrometres in length, approaching the spatial resolution of the X-ray tomography. Therefore the volume fractions derived from neutron diffraction and SEM are more accurate. The data in Fig. 3 indicate that during thermal shock processing ~8% (by volume) of the β-Zr dendrites were converted into the two new phases; this is in contrast to what was reported by Lee *et al.*^13^ who argued that β-Zr does not change when subjected to a stable high temperature (600 °C), while the amorphous matrix was completely crystallised into new crystalline phases. The sample thermally shocked to above 700 °C was completely melted and regained its as-cast microstructure after solidification. However, the volume fraction of β-Zr in this sample (60.97%) was lower than in the as-cast sample (69.21%), because fast cooling rate after thermal shocking resulted in more amorphous matrix; this confirms that the fast heating using electric current followed by the relative fast cooling can retain the designed composite microstructure^11^.

Previous studies^17^,^18^ of isothermal annealing have found that quasicrystals nucleate (and then grow into nanocrystals) within the glassy matrix. However, we have found that nucleation and growth occur predominantly at dendrite-matrix interfaces. Figure 1e–g show the typical microstructure of the transition region (marked by an oval in Fig. 1a) between the as-cast and the thermally-shocked region for the 650 °C sample. It indicates that the majority of the new Zr_2_Cu and ZrBe_2_ crystalline phases nucleated at the interface and grew together into the amorphous matrix in a form of eutectic growth^4^. Some of the Zr_2_Cu and ZrBe_2_ phases (Fig. 1h and i) were also found to nucleate directly from the amorphous matrix and grow into the surroundings, similar to those found in most nanocrystallisation studies^9^,^19^,^20^. However, in our study, the evidence indicates that the dendrite-amorphous matrix interface is the dominant nucleation site for the new crystalline phases.

## Conclusion

The 3D nano and microstructures of DH3 at different thermal shock conditions were studied and characterised using X-ray microtomography and FIB-SEM nanotomgraphy. We found that the ductile β-Zr crystalline dendrites are interlocked 3D structures with complex morphology of a few hundreds of micrometres. They are not the “globular” particles of a few to tens of microns in length, as previously inferred from 2D imaging. The amorphous to crystalline transition at the interface under thermal shock by applying electric current is very different to that occurred in isothermal heating conditions. The large difference in electric resistivities between the amorphous matrix and the crystalline dendrites results in differential heating across the amorphous-crystalline interface, which leads to the nucleation of new crystalline phases (ZrB_2_ and Zr_2_Cu) preferably at the interface, rather than within the amorphous matrix. They grew concurrently to form 3D eutectic networks as revealed by using FIB-SEM nanotomography.

## Methods

DH3 Alloy ingots with a composition of Zr_39.6_Ti_33.9_Nb_7.6_Cu_6.4_Be_12.5_ were made by arc melting a mixture of Ti, Zr, Nb, Cu, and Be with purities ≥99.9% under a Ti-gettered argon atmosphere. The alloys were remelted five times before being cast into a copper mold to form button ingots of 45 mm diameter. Rod bars of ∅6 mm × 30 mm were produced from the ingots via electrical discharge machining. To measure temperature, S-type thermocouples of ∅ 0.2 mm were spot-welded onto the surface at the midpoint of the long edge of the bar samples. A Gleeble 3500 thermo-mechanical simulator and Cu electrodes were used to clamp the rods at both ends and high density electric currents were then applied to thermally shock the rods to preset temperatures of 600 and 700 °C at an initial heating rate of ~500 °C/s from room temperature, and then held for 5 s before cooling down. The heating rate was chosen based on analyses of the results reported by Wang^21^ and Schroers^19^. The target temperatures were chosen because the glass transition temperature, T_g_ of the DH3 is measured at ~350 °C, and it starts to melt at ~800 °C at a heating rate of ~0.8K/s^13^.

The microstructures of the as-cast and thermally shocked samples were characterised using a Zeiss Evo60 scanning electron microscope operating at 20 kV. The chemical compositions of phases were analysed using energy disperse X-ray spectroscopy (EDX) within the SEM.

Neutron diffraction data of the as-cast and thermally shocked region of a rod bar rapidly heated up to 650 °C are shown in Fig. 1a. The data were collected using the General Materials Diffractometer (GEM) beamline at ISIS Neutron and Muon Source, UK^22^. The beam size was 15 × 15 mm, and time of flight diffraction patterns were collected in 50 minutes to give sufficient counting statistics. The diffractograms were analysed and fitted by the Rietveld method implemented in the EXPGUI^23^ and GSAS programs^24^.

Synchrotron X-ray microtomography experiments were conducted at beamline I13-2 of Diamond Light Source, UK^25^. Samples were ground and polished to needle-shapes with ~70 µm tip diameters. From the EDX analyses and published data^6^, the compositions of dendritic and amorphous phases were determined to be Zr_44.0_Ti_40.5_Nb_14.0_Cu_1.5_ and Zr_32.0_Ti_18.0_Nb_3.0_Cu_10.0_Be_37.0_ respectively. We used the Centre for X-ray Optics’ X-Ray Interactions With Matter online calculator^26^,^27^ to calculate X-ray transmission as a function of energy for the two phases. A relatively large difference (10%) in transmission for the two phases was predicted to be at 17.3 keV, and a monochromatic beam of this energy was therefore used to give reasonable contrast. In order to optimize phase contrast, various propagation (sample-to-scintillator) distances were trailed^28^, and 30 mm was chosen. Tomography data were acquired using a pco.edge 5.5 detector (PCO AG, Germany) coupled to a 500 μm thick CdWO_4_ scintillator and visual optics with 8 × magnification. This provided an effective pixel size of 0.81 μm, and a field of view of 2.1 × 1.8 mm. For each tomography scan, 2,001 X-ray projection images were acquired over 180° degrees, with exposure times of 1.9 seconds. Reconstruction was performed with the tomographic reconstruction module of DAWN v1.7^29^,^30^ and the computing cluster at Diamond Light Source. Noise reduction, tophat-based segmentation and volume fraction quantifications were performed with AVIZO v8.0 (FEI, USA). The secondary dendrites’ arm spacing and tip and root curvature radii were measured using the Principal Curvature Plugin (2D/3D)^31^ in ImageJ^32^ based on the cross section contour line for typical secondary dendrites acquired with AVIZO.

FIB-SEM sectioning was carried out using a LYRA3 XM (Tescan s.r.o., Brno, Czech Republic). 50 nm layers were removed by FIB, and SEM imaging was performed on each newly exposed surface. The total FIB milling depth was set to 20 μm, the cross-sectional area to 20 × 20μm, and 170 sectional images were obtained. This was to ensure that at each milling step, a complete section through the β-Zr, Zr_2_Cu and ZrBe_2_ phases was achieved. Precise alignment of the ion and electron beams was maintained to stabilise the centres of successive images and to avoid electron image drift or “jitter”. To study the microstructure of the Zr_2_Cu and ZrBe_2_ intermetallic phases, higher resolution SEM images were acquired. For each image frame, the effective pixel size was set to 25 × 25 nm, and a matrix of 676 × 468 pixels was used. Periodic noise was removed by masking out the noise spots found in the Fourier space images obtained from the fast Fourier transformation of the raw images using ImageJ; AVIZO v8.0 (FEI, USA) was used for further noise reduction, 3D rendering with tophat-based segmentation.

## Additional Information

**How to cite this article**: Zhang, W. *et al.* Multi-scale Characterisation of the 3D Microstructure of a Thermally-Shocked Bulk Metallic Glass Matrix Composite. *Sci. Rep.* **6**, 18545; doi: 10.1038/srep18545 (2016).

## Supplementary Material

Supplementary Video S1

Supplementary Video S2

Supplementary Information
