# Supplementary material for: Multi-scale Characterisation of the 3D Microstructure of a Thermally-Shocked Bulk Metallic Glass Matrix Composite
Source: Sci Rep. 2016 Jan 4;6:18545. doi: 10.1038/srep18545 (PMC4698588; doi:10.1038/srep18545)
Supplement: Supplementary Information [file srep18545-s3.pdf]

Multi-scale Characterization of the 3D Microstructure of a Thermally-Shocked Bulk Metallic Glass Matrix Composite

Wei Zhang[a], Andrew J. Bodey[b], Tan Sui [c], Winfried Kockelmann[d], Christoph Rau[b], Alexander M. Korsunsky[d], Jiawei Mi[a*]

[a]School of Engineering, University of Hull, Hull, HU6 7RX, East Yorkshire, UK
[b]Diamond Light Source, Oxfordshire, OX11 0DE, UK
[c] Multi-Beam Laboratory for Engineering Microscopy (MBLEM), Department of Engineering Science, University of Oxford, Parks Road, Oxford， OX1 3PJ, UK
[d]ISIS Neutron and Muon Source, Rutherford Appleton Laboratory, Oxfordshire, OX11 0QX, UK

The legend information for the Supplementary Video S1 and S2 are described below:

Supplementary Video S1:

Title of the video:

β-Zr dendrites of as-cast DH3 (Zr-Ti based bulk metallic glass matrix composite).

Description of the video:

The video shows the 3D morphologies of a cluster of β-Zr dendrites. Firstly, the dendrites without amorphous matrix were shown. Secondly, a single dendrite was chosen from the cluster to show its characteristics in all directions. Thirdly, the amorphous matrix without the cluster of β-Zr dendrites was shown to indicate that the amorphous matrix is actually a continuous network, in which the β-Zr dendrites cluster is accommodated. Finally, the stack of 2D sections was shown to demonstrate how the 3D structures were generated via 2D X-ray tomograms.

Supplementary Video S2:

Title of the video:

Zr_2Cu and $ZrBe_2$ intermetallic phases of thermally shocked DH3 (Zr-Ti based bulk metallic glass matrix composite)

Description of the video:

The video shows the 3D structure of the eutectic colony formed by the Zr_2Cu (the green phase) and $ZrBe_2$ (the yellow phase) intermetallic phases, where the β-Zr dendrites are hidden. The stack of the 2D sections was shown to demonstrate how the 3D structures were generated via 2D sectional SEM images, in which β-Zr dendrites are displayed with uniform grey background in those 2D sections.
